# Not All Painless Jaundice in the Elderly Is Primary Pancreatic Cancer

**DOI:** 10.1155/crom/2124659

**Published:** 2026-04-07

**Authors:** Manas Pustake, Virali Gulla, Ritwik Dey, Edwin Mendoza, Bhaswanth Bollu, Yagnapriya Chirrareddy, Lakshmi Kattamuri, Ramin Nassiry, Abhizith Deoker

**Affiliations:** ^1^ Department of Internal Medicine, Texas Tech University Health Science Center, El Paso, Texas, USA

## Abstract

Painless jaundice in elderly patients typically suggests primary pancreaticobiliary malignancy, but metastatic disease to the periampullary region represents an uncommon yet important differential diagnosis that can present with identical clinical features. We present a case of a 65‐year‐old woman with a history of Stage IIIB endometrial adenocarcinoma with known pelvic and mesenteric metastases, previously treated with salvage chemotherapy, who presented with 3 days of progressive painless jaundice, dark urine, and a year‐long history of worsening diarrhea with significant weight loss. Laboratory evaluation revealed a cholestatic pattern of liver injury (total bilirubin 8.1 mg/dL and alkaline phosphatase 862 U/L), and imaging demonstrated new intra‐ and extrahepatic biliary ductal dilation with a spiculated duodenal mass infiltrating the ampulla and pancreatic head, along with bilateral hydroureteronephrosis from pelvic disease. Endoscopic retrograde cholangiopancreatography confirmed an infiltrative ampullary mass with successful biliary stent placement for decompression, resulting in improvement of hyperbilirubinemia. This case demonstrates that metastatic endometrial carcinoma can present as painless obstructive jaundice through duodenal and ampullary involvement, closely mimicking primary pancreatic adenocarcinoma both clinically and radiographically. Recognition of atypical metastatic patterns is essential for appropriate oncologic management, as treatment strategies and prognosis differ significantly between primary pancreaticobiliary malignancy and metastatic disease.

**Summary**

Painless jaundice in patients with prior malignancy should not be presumed to be primary pancreatic cancer. Endometrial carcinoma can rarely metastasize to the periampullary region, mimicking pancreatic adenocarcinoma clinically and radiographically, making multidisciplinary evaluation essential for accurate diagnosis and appropriate management.

## 1. Introduction

Endometrial carcinoma is one of the most common gynecologic malignancies in Western countries [1]. It is traditionally classified into two pathophysiologic types. Type I includes endometrioid and mucinous carcinomas. These are typically estrogen‐dependent and have a better prognosis. Type II includes serous, clear cell, undifferentiated carcinoma, and carcinosarcoma. These are estrogen‐independent and show early metastatic tendency. FIGO histologic grading is based on the proportion of solid nonglandular/squamous growth: Grade 1 (≤ 5%), Grade 2 (6%–50%), and Grade 3 (> 50%) [1].

It generally has a favorable prognosis, as most patients present with early‐stage disease. Metastasis typically occurs in advanced stages, involving local sites such as pelvic or para‐aortic lymph nodes, the vagina, and the adnexa or distant organs, including inguinal nodes, the peritoneum, lungs, the liver, and bones [2]. The liver is the most commonly affected intra‐abdominal organ. The duodenum is an uncommon site for metastasis and is rarely involved in endometrial carcinoma [3]. The metastatic dissemination pathway to the duodenum remains unclear, although retrograde lymphatic spread from para‐aortic lymph nodes has been proposed [3].

Obstructive jaundice can result from both benign and malignant causes, with pancreatic cancer being the most common neoplastic etiology [4]. Common primary malignancies that may metastasize to the biliary tree include cancers of the colon, breast, lung, stomach, and melanoma [4]. Metastasis of endometrial carcinoma to the pancreas or periampullary region, causing obstructive jaundice or pancreatitis, is extremely rare. Only a handful of cases have been reported in the literature, making this an uncommon and clinically significant presentation. Here, we present a rare case of endometrial carcinoma metastasizing to the duodenum, resulting in painless obstructive jaundice.

## 2. Case Presentation

A 65‐year‐old woman with a past medical and oncologic history of endometrioid endometrial adenocarcinoma FIGO Grade 1, Stage IIIB (cervical stroma and parametrial and adnexal involvement) status post‐total abdominal hysterectomy, bilateral salpingo‐oophorectomy, and sentinel lymph node dissection (4 years back) presented with a 3‐day history of progressive jaundice and a year‐long history of worsening diarrhea. Her oncologic history was notable for metastatic recurrence with pelvic, mesenteric, and right pubic tubercle implants, managed with salvage chemotherapy (doxorubicin liposomal, last administered a month before presentation, Cycle 9), and complicated by prior neutropenia. She also had a history of pulmonary embolism (on apixaban maintenance) and an open gastrojejunostomy (a year ago) for malignant gastric outlet obstruction.

The patient reported an insidious onset of yellowing of the skin and sclera, dark urine, and persistent, foul‐smelling, sometimes fatty diarrhea, with significant unintentional weight loss (15–20 lbs in 1 month). She denied abdominal pain, nausea, vomiting, or fever. Physical examination was remarkable for scleral icterus, jaundice, and mild hepatomegaly but was otherwise unremarkable.

Laboratory evaluation demonstrated a cholestatic pattern of liver injury (total bilirubin 8.1 mg/dL, direct bilirubin 5.6 mg/dL, ALP 862 U/L, AST 142 U/L, and ALT 206 U/L), mild leukocytosis, and hypokalemia. Imaging (CT abdomen/pelvis, MRCP, and abdominal ultrasound) demonstrated interval development of intra‐ and extrahepatic biliary and pancreatic ductal dilation, a spiculated mass in the second portion of the duodenum infiltrating the ampulla and head of the pancreas, and bilateral hydroureteronephrosis (right > left) due to encasement of the distal ureters by pelvic and duodenal masses. No new hepatic or distant metastases were identified (Table 1).

**Table 1:** Diagnostic workup.

Test category	Specific tests	Results
Laboratory tests	CBC with differential	Leukocytosis (WBC 11.08 × 10^3^/*μ*L), anemia (Hb 10.2 g/dL), and macrocytosis (MCV 103 fL)
Laboratory tests	CMP	Elevated total/direct bilirubin (8.1/5.6 mg/dL), ALP (862 U/L), AST (142 U/L), ALT (206 U/L), hypoalbuminemia (3.8 g/dL), and hypokalemia (2.3 mmol/L)
Laboratory tests	PT/INR/APTT	PT 15 s, INR 1.2, and APTT 20.8 s
Laboratory tests	Troponin/NT‐proBNP	Mildly elevated (troponin 17 ng/L and NT‐proBNP 710 pg/mL)
Laboratory tests	Infectious workup	Negative *C. difficile*, viral panel, stool O&P, and hepatitis serologies
Imaging studies	Ultrasound abdomen	Dilated CBD (11.65 mm), hydropic gallbladder, and right hydronephrosis
Imaging studies	CT abdomen/pelvis	Duodenal mass infiltrating ampulla/head of pancreas, intra/extrahepatic biliary and pancreatic ductal dilation, and bilateral hydronephrosis (right > left)
Imaging studies	MRCP	Confirms biliary and pancreatic ductal dilation, hydropic gallbladder, and suspicious for malignancy
Imaging studies	MRI abdomen	Bilateral hydronephrosis, right > left; distal ureters not fully imaged
Endoscopic procedures	ERCP	Infiltrating mass at the major papilla, moderately dilated biliary tree, and successful biliary stent placement
Endoscopic procedures	Upper endoscopy	Likely malignant duodenal mass, benign‐appearing esophageal stenosis, and intact gastrojejunostomy
Histopathology	Biopsy	Deferred due to bleeding risk; clinical/imaging correlation supports metastatic endometrial carcinoma

The patient underwent ERCP with placement of a covered metal biliary stent for decompression. Endoscopic evaluation confirmed an infiltrative, likely malignant duodenal mass at the ampulla, but biopsy was deferred due to recent anticoagulation and bleeding risk. Consequently, immunohistochemical (IHC) studies (CK7, CK8/18, CK19, and Müllerian markers) could not be performed. Postprocedure, her hyperbilirubinemia and transaminitis improved. Infectious workup, including *Clostridioides difficile*, viral panel, and stool O&P, was negative. Diarrhea was attributed to pancreatic exocrine insufficiency secondary to ampullary and pancreatic head involvement.

During hospitalization, the patient experienced a single episode of vaginal or rectal bleeding while on heparin (apixaban was switched to heparin in the hospital), prompting cessation of anticoagulation and close monitoring. Hemoglobin stabilized without further intervention. Urology was consulted for bilateral hydronephrosis; given preserved renal function and absence of symptoms, no acute intervention was recommended.

Multidisciplinary review (GI, general surgery, gynecologic oncology, hematology/oncology, urology, and palliative care) concluded that the duodenal/ampullary mass represented progression of metastatic endometrial carcinoma rather than a new primary pancreatic malignancy. Given resistance to cytotoxic chemotherapy and disease progression, the consensus was to discontinue further cytotoxic or immune therapy and transition to a palliative approach, with outpatient follow‐up for symptom management.

## 3. Discussion

The differential diagnosis for an ampullary mass includes primary periampullary carcinoma, distal cholangiocarcinoma, and ampullary villous adenoma. Definitive diagnosis requires histopathology and IHC to confirm metastatic disease and identify the primary source [5].

Periampullary metastases are rare, with most reports limited to single cases or small series. Primary tumors that commonly metastasize to this region include renal cell carcinoma, lung carcinoma, gastrointestinal cancers, breast carcinoma, lymphoma, and melanoma [2]. Endometrial carcinoma metastasizing to the periampullary area is exceptionally rare, with only two or three cases reported. We report a 65‐year‐old female with Stage IIIB endometrial carcinoma diagnosed 4 years ago and treated with standard therapy, who developed malignant gastric outlet obstruction 1 year ago and now presents with periampullary metastasis. The interval between primary diagnosis and periampullary involvement is highly variable, and prognosis is generally poor, highlighting the diagnostic and therapeutic challenges of such unusual metastatic spread. Secondary (metastatic) ampullary tumors often present with nonspecific symptoms similar to primary ampullary cancers, including abdominal discomfort, jaundice, pruritus, and changes in stool or urine color [6].

Diagnostic imaging for malignant distal biliary obstruction, including abdominal ultrasound, CT, MRCP, ERCP, and endoscopic ultrasound, helps in detecting lesions and staging but cannot reliably distinguish primary from metastatic disease [2]. Endoscopically, they often appear as polypoid, irregular, friable, or ulcerated masses, making them indistinguishable from primary lesions [6]. Therefore, definitive diagnosis depends on histopathological evaluation and IHC analysis to confirm the metastatic nature and identify the primary source [7]. IHC testing plays a critical role in distinguishing metastatic from primary disease and differentiating synchronous from metachronous malignancies. Key markers include CK7, which is characteristically positive in both endometrial and pancreatobiliary tumors; CK19, which supports a biliary or pancreatic origin; and CK8/18, a broad epithelial marker. Endometrial carcinoma typically demonstrates a CK7+/CK20− immunoprofile, a pattern that overlaps with pancreatobiliary‐type ampullary adenocarcinoma yet helps exclude intestinal‐type tumors and other metastatic etiologies [8]. In our case, biopsy and thus IHC could not be performed due to the risk of bleeding. Diagnosis was therefore established through careful integration of clinical history, multimodality imaging, and multidisciplinary team consensus.

Primary periampullary carcinoma, including ampullary and distal bile duct cancers, is often managed with curative intent when resectable, typically via pancreaticoduodenectomy. By contrast, management of periampullary metastases is usually palliative [6]. Surgical resection, such as a Whipple′s procedure, may be considered in select cases but is often not feasible due to advanced disease or distant metastases [7]. Most patients are managed palliatively with biliary drainage or stenting to relieve obstruction and improve symptoms, followed by systemic chemotherapy when appropriate [2]. In our case, the patient underwent ERCP with placement of a covered metal biliary stent to relieve obstruction. In concern of disease progression and resistance to cytotoxic therapy, further systemic or immunotherapy was discontinued, and the patient was transitioned to a palliative care approach (Table 2).

**Table 2:** Comparison of primary versus metastatic periampullary tumors.

Feature	Primary periampullary tumors	Metastatic periampullary tumors
Epidemiology and origin		
Frequency	Common (85%–95% of periampullary masses)	Rare (5%–15% of periampullary masses)
Origin	Arise locally from periampullary organs	Spread from a distant primary cancer site
Common types	• Pancreatic adenocarcinoma (60%–70%) • Ampullary carcinoma (20%) • Distal cholangiocarcinoma (10%) • Duodenal adenocarcinoma (10%)	• Renal cell carcinoma • Melanoma • Breast carcinoma • Lung carcinoma • Gynecologic malignancies

Clinical presentation		
Age at presentation	60–70 years (median)	Variable, depends on the primary tumor
Key symptoms	• Painless jaundice (70%–90%) • Weight loss (60%–80%) • New‐onset diabetes (15%–20%) • Courvoisier′s sign (25%–50%)	• Painless jaundice (similar) • Often with systemic symptoms (e.g., fatigue) • Symptoms from other metastatic sites
Patient history	De novo presentation	History of known primary malignancy (80%–90%)
Timeline	N/A	Typically 2–5 years after primary diagnosis

Diagnostic features		
Tumor markers	• CA 19‐9 elevated (70%–90%) • CEA elevated (40%–60%)	• Markers specific to the primary tumor • CA 19‐9 may be elevated, but is usually lower • Mixed marker pattern
Imaging (CT/MRI)	• Hypoattenuating mass • “Double duct sign” common • Vascular encasement frequent • Regional lymphadenopathy	• Multiple discrete masses more common • Less likely “double duct sign” • Other metastatic sites often visible
Endoscopic findings	• Friable, irregular ampullary mass • Single dominant lesion	• Multiple periampullary nodules possible • Submucosal infiltration pattern
Histopathology	• Adenocarcinoma most common • Pancreatic: Infiltrative glands and desmoplastic stroma	• Morphology consistent with primary tumor • Immunohistochemistry is crucial (e.g., PAX8 for RCC)

Management and prognosis		
Primary management	• Surgical resection (Whipple′s procedure) if feasible • Neoadjuvant/adjuvant chemotherapy • Radiation in select cases	• Palliative biliary drainage • Systemic therapy based on the primary tumor • Rarely surgical candidates
Prognosis	• Pancreatic: Poor (median survival 6–11 months) • Ampullary: Better (median survival 30–50 months)	Generally poor (median survival 3–9 months)
Diagnostic approach	• EUS with FNA • ERCP with biopsy • CA 19‐9, CEA • Staging CT	• Compare with prior imaging and pathology • Immunohistochemistry essential • PET‐CT to find other metastases • Tumor markers based on suspected primary
Key distinguishing features	• No history of other malignancy • Single site of disease • Classic imaging patterns (e.g., double duct sign) • Consistent tumor marker profile	• Known primary malignancy • Multiple sites of disease • Atypical imaging features • Mixed tumor marker pattern

## 4. Conclusion

This case highlights the importance of maintaining a broad differential for painless jaundice in elderly patients, particularly those with a history of malignancy. While primary pancreatic adenocarcinoma is a common etiology, metastatic disease—especially from endometrial carcinoma—can present with similar clinical and radiographic features, including biliary obstruction due to duodenal or ampullary involvement. Recognition of atypical metastatic patterns is critical for appropriate management and prognostication.

## Author Contributions

Manas Pustake, Virali Gulla, Ritwik Dey, Edwin Mendoza, Bhaswanth Bollu, Yagnapriya Chirrareddy, Lakshmi Kattamuri, Ramin Nassiry, and Abhizith Deoker contributed to data acquisition, literature review, manuscript drafting, and critical revision.

## Funding

No funding was received for this manuscript.

## Disclosure

All authors approved the final manuscript and agreed to be accountable for all aspects of the work.

## Ethics Statement

Ethical approval was not required for this case report in accordance with institutional policy.

## Consent

Consent was obtained from the patient for publication of this case report.

## Conflicts of Interest

The authors declare no conflicts of interest.

## Data Availability

Data sharing is not applicable to this article as no datasets were generated or analyzed during the current study.
